# KDM2B regulates inflammation and oxidative stress of sepsis via targeting NF‐κB and AP‐1 pathways

**DOI:** 10.1002/iid3.985

**Published:** 2023-09-20

**Authors:** Xin Li, Xinyu Tian, Dongliang Zhang

**Affiliations:** ^1^ Department of Nephrology Beijing Jishuitan Hospital, Capital Medical University Beijing China

**Keywords:** AP‐1, cell proliferation, inflammation, KDM2B, NF‐κB, oxidative stress, sepsis

## Abstract

**Backgrounds:**

The kidney is an easily affected organ with sepsis which is a main underlying cause of acute kidney injury (AKI). Histone‐modifying lysine‐specific demethylase 2B (KDM2B) is involved in numerous pathological processes, such as cell senescence and tumor development. However, the role of KDM2B in sepsis‐induced AKI is unclear.

**Objects:**

To investigate the role of KDM2B on cell viability, inflammation and oxidative stress of sepsis‐associated AKI, and the involved signaling pathways.

**Methods:**

An AKI model in vitro was established through lipopolysaccharide (LPS)‐induction in HK‐2 cells. Western blots were performed to evaluate the expression of KDM2B, cyclooxygenase 2 (COX2), inducible nitric oxide synthase (iNOS), p65, c‐Jun and c‐Fos, as well as p65 phosphorylation. Cell viability was measured using CCK‐8 kit. ELISA was performed to analyze the production of layered double hydroxide (LDH), tumor necrosis factor (TNF)‐α, interleukin (IL)‐1β, IL‐18, vascular cell adhesion molecule‐1 (VCAM‐1), superoxide dismutase (SOD), malondialdehyde (MDA), glutathione (GSH), and H_2_O_2_. The qPCR was used to evaluate the transcription level of TNF‐α, IL‐1β, IL‐18, and VCAM‐1.

**Results:**

KDM2B knockdown alleviated LPS‐induced cytotoxicity, decreased LDH release, and improved cell viability. KDM2B knockdown reduced concentration of inflammation‐related molecules including TNF‐α, IL‐1β, IL‐18, and VCAM‐1, and inhibited their transcription. Moreover, KDM2B knockdown promoted the quantity of SOD and GSH, while declined the production of MDA, H_2_O_2_, COX2, and iNOS. Further, KDM2B played a role in LPS‐induced HK‐2 cell injury by activating nuclear factor κB (NF‐κB) and activator protein 1 (AP‐1) pathways.

**Conclusion:**

KDM2B knockdown reduced cytotoxicity, inflammation and oxidative stress in LPS‐induced AKI via inhibiting NF‐κB and AP‐1 pathways, indicating KDM2B may be a promising therapeutic target for the treatment of sepsis‐associated AKI.

## 1 INTRODUCTION

Sepsis commonly occurs in the kidney, which is one kind of organ dysfunction resulting from an adverse host response to infection. It always leads to a condition called sepsis‐associated acute kidney injury (AKI), which increases morbidity and mortality from sepsis.^1^ The reported incidence of AKI in septic patients ranges from 22% to 53%. Even if AKI patients survive, the likelihood of chronic kidney disease is greatly increased. It has been reported that damage to renal tubular epithelial cells (RTEC) is the main underlying cause of AKI.^2^ However, the molecular mechanisms of RTEC injury in AKI are not fully elucidated.

Inflammation plays a crucial role in sepsis development and progression. During infection, the body's immune system responds by releasing various inflammatory molecules, such as cytokines, chemokines, and prostaglandins, to help fight off the invading pathogens.^3^ In sepsis, however, the immune response becomes dysregulated, leading to excessive and uncontrolled inflammatory responses throughout the body. This widespread inflammation can damage tissues and organs, leading to the progression of sepsis.^4^ To manage sepsis, it is necessary to address the underlying infection, and control the inflammatory response.

KDM2B belongs to JmjC domain‐containing histone demethylase family. KDM2B has been shown to be involved in a variety of fundamental biological and pathological processes, such as cell cycle, senescence, and tumor development.^5^ KDM2B regulates choline kinase‐α at the session of neuronal differentiation and maintains the undifferentiated stage of neuroblasts.^6^ KDM2B also elevated interleukin (IL‐6) production and activated inflammatory responses through gene‐specific transcriptional initiation.^7^ In addition, KDM2B induces activator protein 1 (AP‐1) transcriptional activity via SKP1‐CUL1‐F‐box (SCF) E3 ubiquitin ligase complex, mediating Kaposi sarcoma‐associated herpesvirus infection.^8^ However, the role of KDM2B in sepsis‐induced RTEC injury is unclear.

In this study, the cell viability, cytotoxicity, inflammation, and oxidative stress were investigated on lipopolysaccharide (LPS)‐induced AKI model with KDM2B knockdown, while the regulation of nuclear factor κB (NF‐κB) and AP‐1 pathway was also analyzed, which serve to conclude that KDM2B knockdown reduced cytotoxicity, inflammation, and oxidative stress in LPS‐induced AKI via inhibiting NF‐κB and AP‐1 pathways.

## 2 MATERIALS AND METHODS

### 2.1 Reagents and antibodies

Dulbecco's modified Eagle's medium (DMEM) and fetal bovine serum (FBS) were purchased from Gibco. LPS was obtained from MilliporeSigma (L3012). Antibodies against KDM2B (ab234082; dilution 1:500), cyclooxygenase 2 (COX2) (ab300668; dilution 1:2000), and inducible nitric oxide synthase (iNOS) (ab210823; dilution 1:200) were purchased from Abcam. Antibody against phosphorylated p65 (p‐p65) was obtained from Cell Signaling Technology. Antibodies against p65 (663535‐1‐Ig; dilution 1:3000), c‐Jun (66313‐1‐Ig; dilution 1:2000), c‐Fos (66590‐1‐Ig; dilution 1:2000), GAPDH (60004‐1‐Ig; dilution 1:5000) and horseradish peroxidase (HRP)‐conjugated goat anti‐mouse IgG (SA00001‐1; dilution 1:5000) were purchased from ProteinTech. Enzyme‐linked immunosorbent assay (ELISA) kits of tumor necrosis factor (TNF)‐α (KE00154), IL‐1β (KE00021), IL‐18 (KE00193), and vascular cell adhesion molecule‐1 (VCAM‐1) (KE00163) were obtained from ProteinTech. ELISA kits of layered double hydroxide (LDH) (MBS720560), superoxide dismutase (SOD) (MBS034842), malondialdehyde (MDA) (MBS263626), glutathione (GSH) (MBS265674), and H_2_O_2_ (MBS9367846) were purchased from MybioSource. Lipofectamine 2000 (11668027), RIPA lysis buffer (89901), si‐KDM2B#1 (s39305), and si‐KDM2B#2 (s39306) were obtained from ThermoFisher. SR11302 was provided by Absin (160162‐42‐5, Purity ≥ 98%). JSH‐23 was purchased from Selleck (S7351, Purity 99.16%). CCK‐8 was purchased from Glpbio. The RNeasy Kits was obtained from QIAGEN (74004). Universal RT‐PCR Kit (RP1100) and 2× SYBR Green PCR Mastermix (SR1110) were purchased from Solarbio.

### 2.2 Cell culture

HK‐2 cells which are epithelial cell line from normal adult human kidney were maintained in DMEM added with 10% FBS in a 37°C incubator supplied with 5% CO_2_. For the AKI model establishment, the HK‐2 cells were treated with 10 μg/mL LPS for 24 h to induce sepsis‐associated AKI.^2^, ^9^ The LPS‐induced HK‐2 cells were treated with 1 μM SR11302 as an AP‐1 inhibitor or JSH‐23 as an NF‐κB inhibitor,^10^ 48 h later, the supernatant was used for cytokines analysis and the cells were collected for western blots analysis.

### 2.3 KDM2B knockdown and overexpression

Human KDM2B coding sequence (Gene ID: 84678) was amplified with primers (Forward: 5′‐ CGCGGATCCACCATGGCGGGTCCGCAAA ‐3′ and Reverse: 5′‐ CGTTTTTGACTCAATCGCGGCCGCATA ‐3′. The PCR fragment was inserted into pcDNA3.1 vector for KDM2B overexpression, the empty vector pcDNA3.1 was the negative control (NC). For KDM2B knockdown, si‐KDM2B#1 and si‐KDM2B#2 were transfected into HK‐2 cells using Lipofectamine 2000, transfection with siNC as the control.^11^ Western blot was used to evaluate the knockdown efficiency.

### 2.4 Cell viability assay

HK‐2 cells were cultured in 96‐well plates followed by KDM2B knockdown or overexpression, respectively. Twenty‐four hours later, the cells were replaced with new medium supplemented with 10 μL CCK‐8 per well and cultured for another 5 days. The absorbance was analyzed at 450 nm wavelength, and the cell viability ratio was calculated.^2^

### 2.5 ELISA

LPS‐induced HK‐2 cells were treated with siKDM2B or with siKDM2B and inhibitors together, 48 h later, the supernatant was collected. The concentration of LDH, TNF‐α, IL‐1β, IL‐18, VCAM‐1, SOD, MDA, GSH, and H_2_O_2_ in cell medium were evaluated using ELISA kits. In brief, 100 μL sample was added into ELISA well and incubated for 2 h followed by 100 μL detected antibody for 1 h. Then each well was reacted with enzyme working reagent, TMB reagent, and terminated by stop solution. The absorbance value was read at 450 nm.^12^

### 2.6 Western blot

The total protein of HK‐2 cells was extracted using RIPA lysis buffer. The lysates were processed for immunoblot with the primary antibodies at 4°C overnight, and then incubated with HRP‐conjugated goat anti‐mouse IgG at room temperature for 1 h. Finally, the target bands were visualized with ECL reagents (Solarbio) using ChemiDoc Imaging system (Bio‐Rad). The relative intensity was measured by ImageJ software and normalized to GAPDH.^13^

### 2.7 Quantitative PCR (qPCR)

Total RNA was isolated using RNeasy kits, and 500 ng RNA was used for reverse transcription (RT) using Universal RT‐PCR Kit. ABI 7500 Real‐Time PCR System was used to do qRT‐PCR with 2 × SYBR Green PCR Mastermix. The copy number (Ct) of target genes was calculated by $2^{-∆∆C_{t}}$, and normalized to β‐actin level.^13^ The PCR primers were listed in Table 1.

**Table 1:** Primers list.

Target	Sequence (5′‐3′)
TNF‐α	CTTTGGGATCCTGGCTGATC
	CTTCGCTCTGCTGCATTTCA
IL‐1β	GGACAGCCCAGGTCAAAGG
	AGTTGACGGACCCCAAAAGAT
IL‐18	ATCGCT TCCTCTCGCAACAA
	TCC AGGTTTTCATCATCTTCAGC
VCAM‐1	AGTGGTGGCCTCCTGAATGG
	CTGTGTCTCCTGTCTCCGCT
β‐actin	GTCTGCCTTGGTAGTGGATAATG
	TCGAGGACGCCCTATCATGG

### 2.8 Quantification and statistical analysis

Statistical analysis was performed by GraphPad Prism 8.0 (Dotmatics). Data are presented as mean ± standard deviation (SD) from three biological replicates, and the differences between any two groups were calculated by unpaired *t* tests. Multiple group comparisons were analyzed with analysis of variance.

## 3 RESULTS

### 3.1 KDM2B knockdown alleviates cytotoxicity in sepsis‐associated AKI

**Figure 1:**
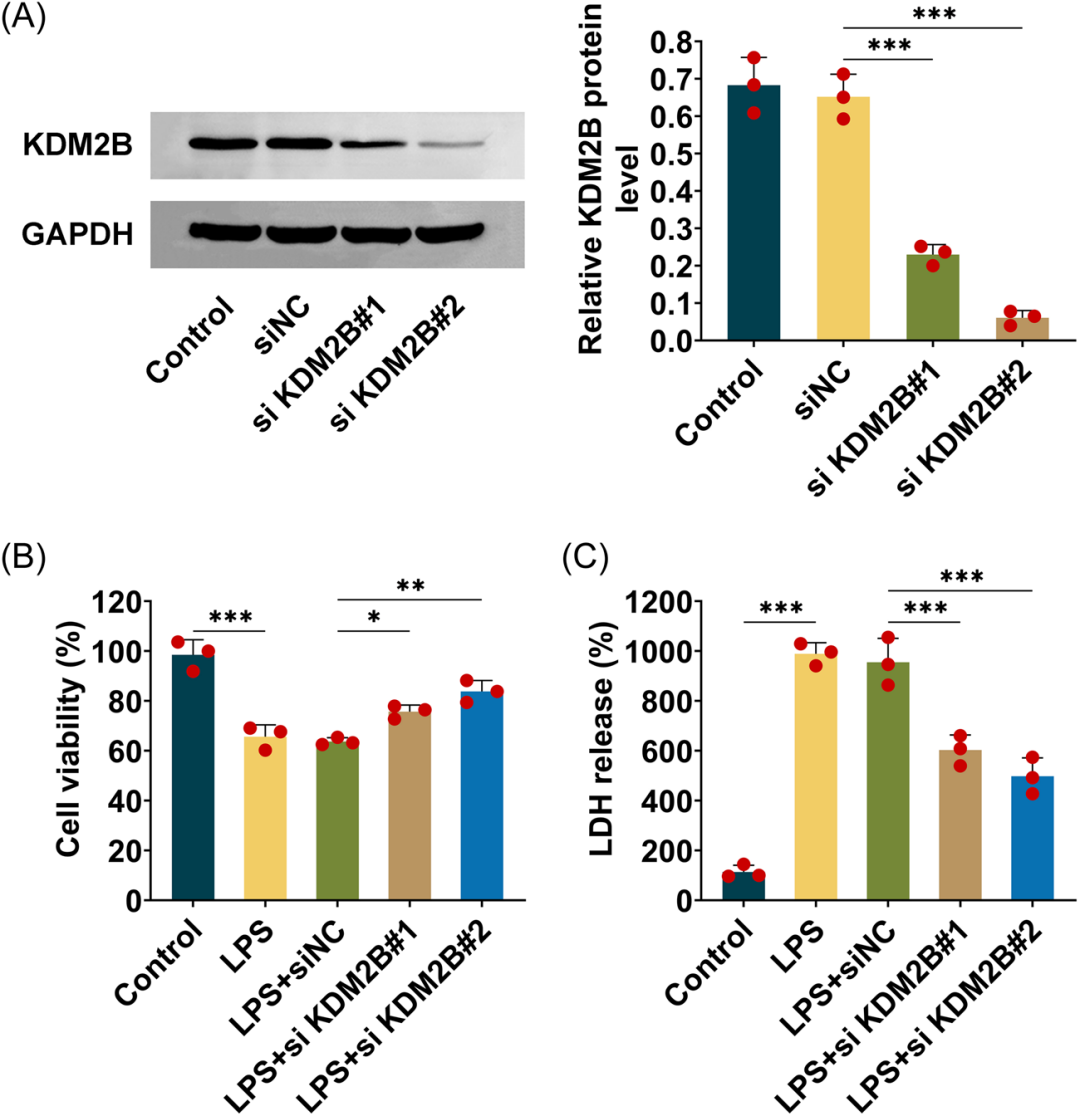
KDM2B knockdown alleviates cytotoxicity in sepsis‐associated AKI. (A) Western blot was performed to evaluate KDM2B expression in HK‐2 cells. (B) The cell viability was evaluated in LPS‐induced HK‐2 cells. (C) The LDH release was measured in LPS‐induced HK‐2 cells transfected with si‐KDM2B#1 or si‐KDM2B#2. The data from three repeated experiments were used for the statistical analysis. Error bar, mean ± SD; *p < .05, **p < .01, ***p < .001. AKI, acute kidney injury; LDH, layered double hydroxide; LPS, lipopolysaccharide.

As is known that AKI induced serious cytotoxicity, to investigate the effect of KDM2B on AKI, HK‐2 cells induced by LPS and followed by KDM2B knockdown was used to analyze the cytotoxicity. First, western blots were conducted to evaluate the expression of KDM2B, the data revealed transfection with si‐KDM2B#1 or si‐KDM2B#2 significantly reduced KDM2B protein level compared with siNC in HK‐2 cells, meaning that si‐KDM2B#1 or si‐KDM2B#2 could be used for KDM2B knockdown (Figure 1A). Next, the cell viability was detected in LPS‐induced HK‐2 cells, the results showed that LPS induction contributed to cell injury with reduced cell viability, however, si‐KDM2B#1 or si‐KDM2B#2 transfection significantly increased the cell viability compared with siNC (Figure 1B). Meanwhile, the LDH concentration in medium was measured which demonstrated that LPS elevated LDH production, but si‐KDM2B#1 or si‐KDM2B#2 transfection significantly decreased the LDH release (Figure 1C). Therefore, KDM2B knockdown alleviates cytotoxicity in HK‐2 cells with LPS‐induced AKI.

### 3.2 KDM2B knockdown reduces inflammation in sepsis‐associated AKI

**Figure 2:**
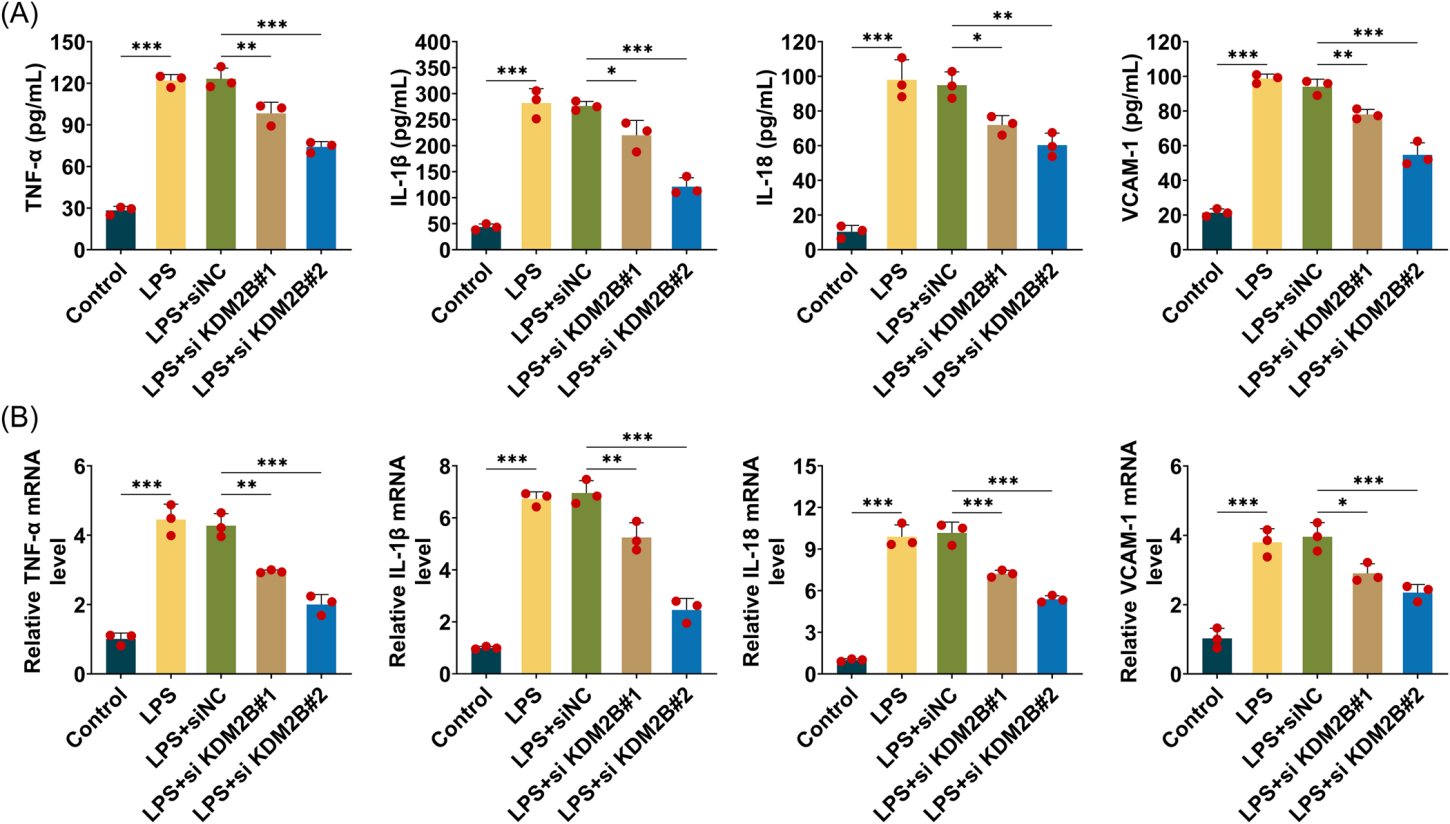
KDM2B knockdown reduces inflammation in sepsis‐associated AKI. (A) ELISA was performed to quantify the concentration of four typical inflammation molecules including TNF‐α, IL‐1β, IL‐18, and VCAM‐1 in LPS‐induced HK‐2 cells. (B) qPCR was used to evaluate the mRNA level of TNF‐ α, IL‐1β, IL‐18, and VCAM‐1. All the experiments were repeated at least three times. Error bar, mean ± SD; *p < .05, **p < .01, ***p < .001. AKI, acute kidney injury.

To investigate the effect of KDM2B on inflammation in AKI model, four typical inflammation molecules such as TNF‐α, IL‐1β, IL‐18, and VCAM‐1 were analyzed in this work. Specifically, ELISA was performed to quantify their concentration in LPS‐induced HK‐2 cells. The results showed all four molecules were increased in LPS‐induced HK‐2 cells, however, their concentration could be declined by si‐KDM2B#1 or si‐KDM2B#2 (Figure 2A). Their mRNA levels were analyzed using qPCR, which revealed that the transcription of TNF‐α, IL‐1β, IL‐18, and VCAM‐1 was significantly weakened in HK‐2 cells with KDM2B knockdown (Figure 2B). The results above suggested that KDM2B knockdown repressed LPS‐induced inflammation in sepsis‐associated AKI.

### 3.3 KDM2B knockdown reduces oxidative stress in sepsis‐associated AKI

**Figure 3:**
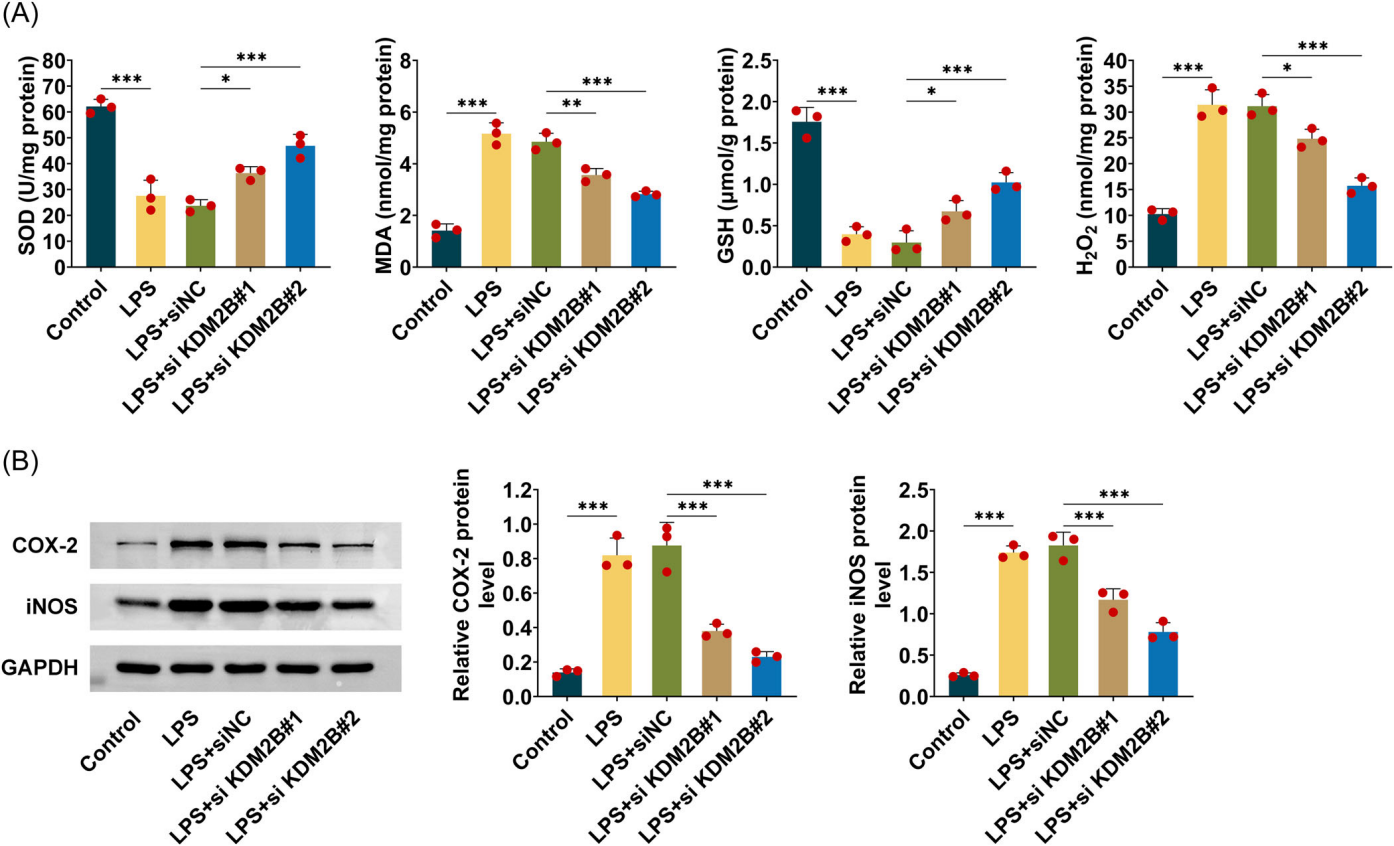
KDM2B knockdown reduces oxidative stress in sepsis‐associated AKI. (A) ELISA was performed to measure the level of SOD, MDA, GSH, and H_2_O_2_ in LPS‐induced HK‐2 cells. (B) iNOS and COX‐2 were measured using western blot in LPS‐induced HK‐2 cells. Three repeated experiments were used for analysis. Error bar, mean ± SD; *p < .05, **p < .01. AKI, acute kidney injury; LPS, lipopolysaccharide.

The effect of KDM2B on oxidative stress was investigated here. ELISA was performed to measure the level of SOD, MDA, GSH, and H_2_O_2_ which are widely accepted markers for oxidative stress.^14^, ^15^, ^16^ For SOD and GSH, their concentrations were downregulated in LPS‐induced HK‐2 cells; however, this kind of downregulation could be alleviated by si‐KDM2B#1 or si‐KDM2B#2, indicating KDM2B knockdown increased the production of SOD and GSH. Correspondingly, the concentration of MDA and H_2_O_2_ were increased in LPS‐induced AKI model, and si‐KDM2B#1 or si‐KDM2B#2 transfection could significantly decline the level of MDA and H_2_O_2_ (Figure 3A). Another two molecules including iNOS and COX‐2 were reported as the typical markers for oxidative stress.^17^, ^18^ In this study, iNOS and COX‐2 were measured using western blot. The data suggested that their expression was elevated in LPS‐induced HK‐2 cells, however, si‐KDM2B#1 or si‐KDM2B#2 could decrease significantly the expression of INOS and COX‐2 (Figure 3B). In sum, KDM2B knockdown reduces LPS‐induced oxidative stress in sepsis‐associated AKI.

### 3.4 KDM2B knockdown inhibits activation of NF‐κB and AP‐1 pathways

**Figure 4:**
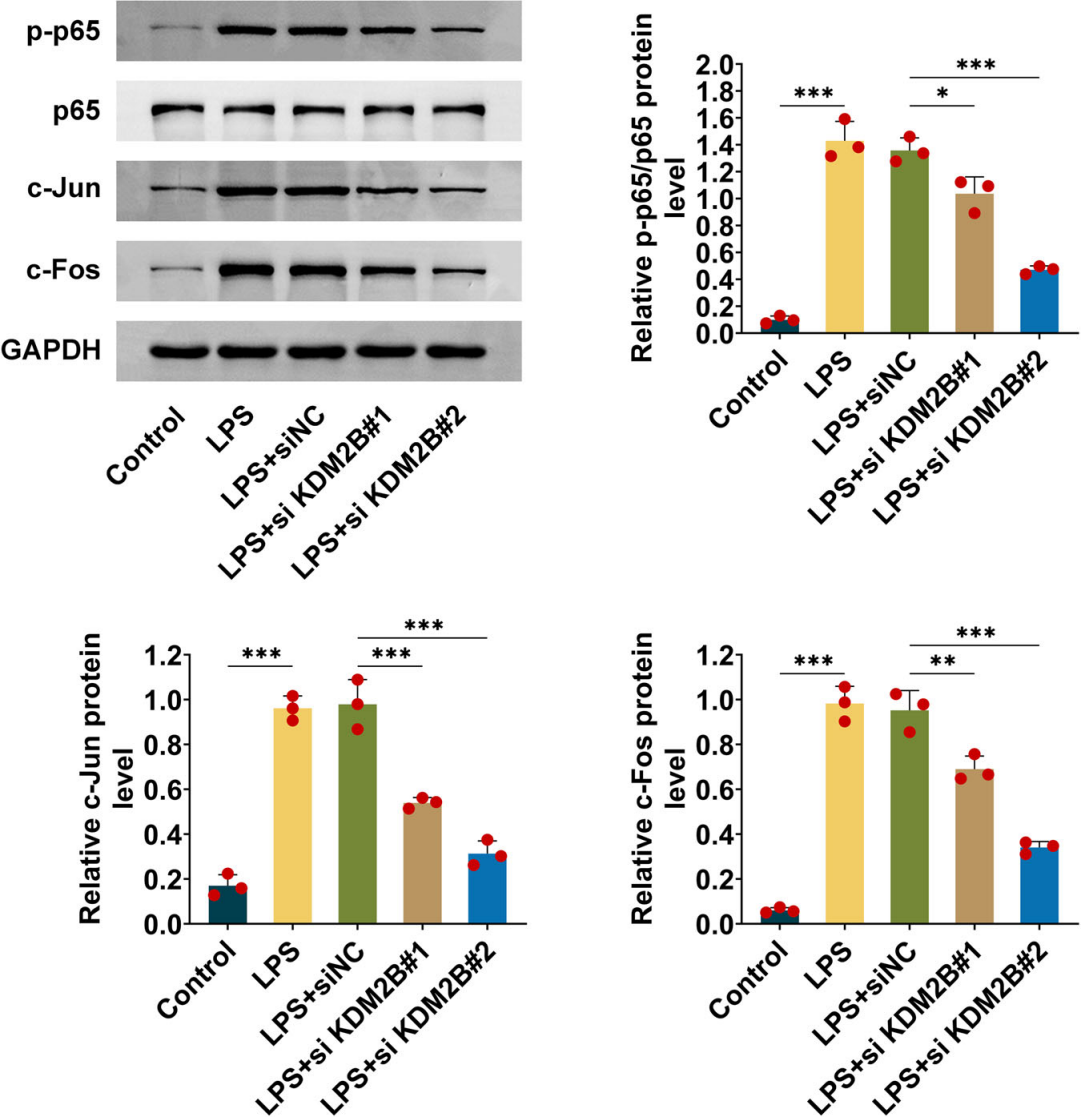
KDM2B knockdown inhibits activation of NF‐κB and AP‐1 pathways. Western blots were performed to evaluate the levels of p65 phosphorylation, the protein expression of c‐Jun and c‐fos in LPS‐induced HK‐2 cells. The data from three independent replications were used for the analysis. Error bar, mean ± SD; *p < .05, **p < .01. LPS, lipopolysaccharide.

It has been known that inflammation is implemented with NF‐κB and AP‐1 signaling pathways. In the present study, several key molecules in these pathways were measured using western blot. The p65 phosphorylation levels were elevated in LPS‐induced AKI, but p65 phosphorylation was declined in si‐KDM2B#1 or si‐KDM2B#2 compared with siNC, which demonstrated that KDM2B knockdown repressed NF‐κB activation. Furthermore, c‐Jun and c‐fos in AP‐1 pathway were also analyzed, the data showed that their expression levels were also suppressed by si‐KDM2B#1 or si‐KDM2B#2 (Figure 4). Thus, KDM2B knockdown inhibits activation of NF‐κB and AP‐1 pathways in LPS‐induced AKI.

### 3.5 KDM2B regulates inflammation and oxidative stress through activating NF‐κB and AP‐1 pathways

**Figure 5:**
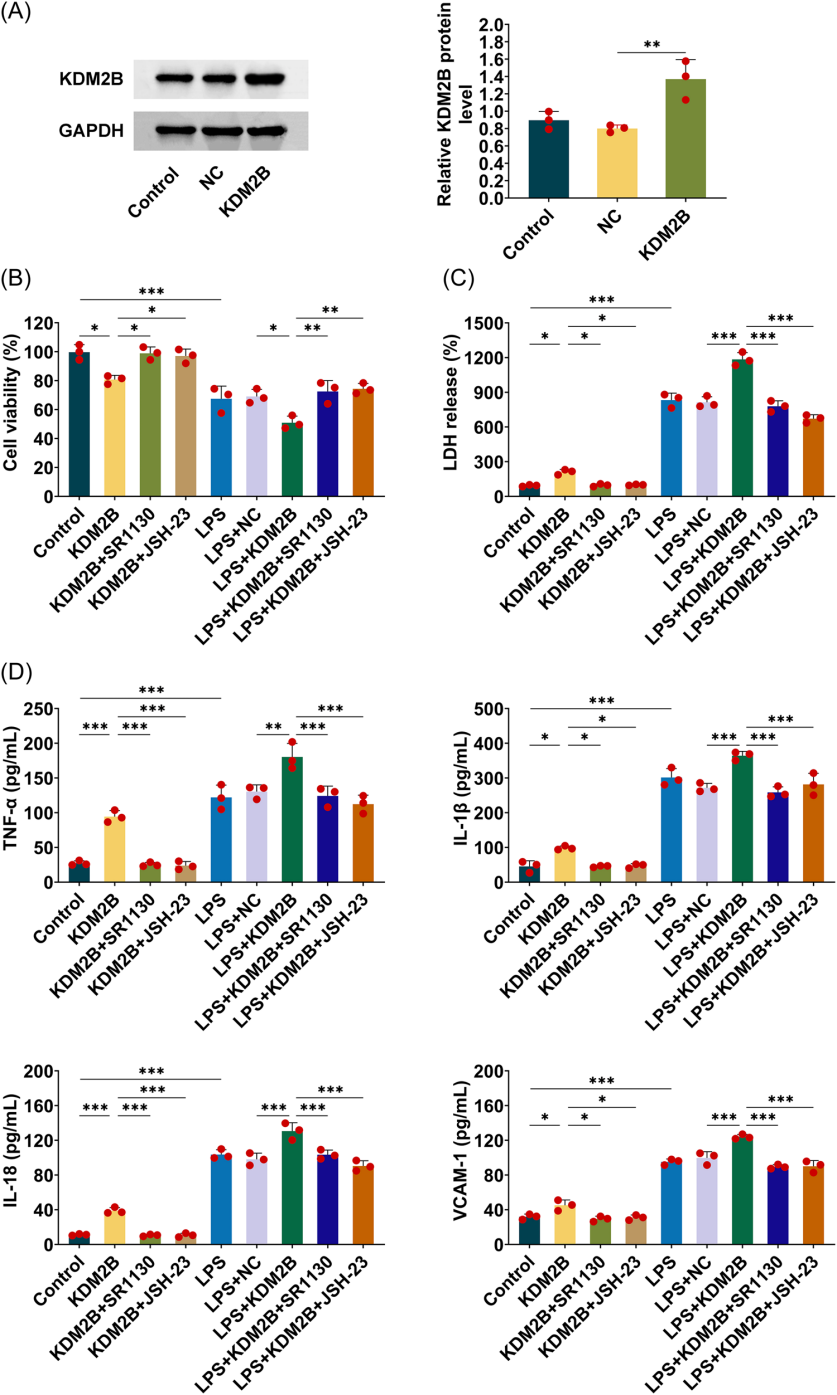
KDM2B regulates inflammation through activating NF‐κB and AP‐1 pathways. (A) Western blot was performed to confirm that KDM2B expression was elevated in HK‐2 cells transfected with KDM2B overexpression plasmid. (B) The cell viability was analyzed in HK‐2 cells. (C) KDM2B overexpression increased LDH production, which was alleviated by SR11302 or JSH‐23 inhibitor. (D) ELISA was used to evaluate the concentration of inflammation factors including TNF‐ α, IL‐1β, IL‐18, and VCAM‐1. Three repeated experiments were used for the statistical analysis. Error bar, mean ± SD; *p < .05, **p < .01, ***p < .001. LDH, layered double hydroxide.

To confirm that KDM2B regulates inflammation and oxidative stress via targeting NF‐κB and AP‐1 pathways, two inhibitors SR11302 and JSH‐23 were used to inhibit NF‐κB and AP‐1 pathways, respectively. Meanwhile, KDM2B overexpression was established by transfecting KDM2B plasmid into HK‐2 cells which increased KDM2B protein level (Figure 5A). KDM2B overexpression declined cell viability, but KDM2B did not affect cell viability once SR11302 or JSH‐23 was supplemented, indicating that the effect of KDM2B overexpression declining cell viability was counteracted by SR11302 or JSH‐23 inhibitor (Figure 5B). Accordingly, KDM2B overexpression increased LDH production, which was alleviated by SR11302 or JSH‐23 inhibitor (Figure 5C). The inflammation factors were also evaluated, the concentration of TNF‐ α, IL‐1β, IL‐18, and VCAM‐1 were increased in KDM2B overexpression groups. However, their level was no longer regulated by KDM2B overexpression if SR11302 or JSH‐23 inhibitor inactivated the NF‐κB and AP‐1 pathways (Figure 5D). Furthermore, the density of SOD and GSH were decreased in KDM2B overexpression groups, but KDM2B overexpression could not regulate SOD or GSH level in HK‐2 cells treated with SR11302 or JSH‐23 inhibitors. KDM2B overexpression elevated MDA and H_2_O_2_ production in LPS‐induced cells, but the activity regulating MDA and H_2_O_2_ was suppressed when NF‐κB and AP‐1 pathways were inactivated by SR11302 or JSH‐23 (Supporting Information: Figure S1A). KDM2B overexpression promoted COX2 and iNOS production, while SR11302 or JSH‐23 inhibitors reduced their production compared with KDM2B overexpression groups (Supporting Information: Figure S1B). Similarly, in LPS‐induced AKI models, the concentrations of COX‐2 and iNOS were elevated in KDM2B overexpressed cells, while their concentrations were decreased in SR11302 or JSH‐23 inhibitors treatment than KDM2B overexpression (Supporting Information: Figure S1C). All the data above demonstrated that KDM2B regulated inflammation and oxidative stress through activating NF‐κB and AP‐1 pathways.

## 4 DISCUSSION

The kidney is an easily affected organ with sepsis which is associated with AKI. The reported incidence of AKI in septic patients ranges from 22% to 53%. Previous studies have shown that damage to RTEC is a main underlying cause of AKI.^2^ KDM2B is involved in numerous pathological processes, for instance, cell senescence and tumor development.^5^ However, the role of KDM2B in sepsis‐induced AKI is unclear. In the present study, an AKI model was established in vitro through LPS induction in HK‐2 cells. LPS induction did reduce cell viability, enhance inflammation and oxidative stress. Meanwhile, two siRNAs (si‐KDM2B#1 and si‐KDM2B#2) successfully contributed to KDM2B knockdown in this LPS‐induced AKI model. The model and KDM2B knockdown provided effective platforms for studying KDM2B's role in regulating sepsis‐associated AKI.

Studies have shown that KDM2B knockdown can alleviate cytotoxicity and improve cell viability in various cell types, including cancer cells, neurons, and cardiomyocytes. For example, KDM2B knockdown in human lung cancer cells led to increased cell viability and decreased cytotoxicity in response to treatment with the chemotherapeutic drug cisplatin.^19^ Furthermore, KDM2B knockdown has been found to reduce LDH release, indicating a decrease in cell damage or death. Moreover, cardiac ischemia/reperfusion injury in mice demonstrated that KDM2B knockdown reduced LDH release and improved heart function, suggesting a protective effect against ischemic injury.^2^, ^20^ Consistently, this work revealed that KDM2B knockdown alleviates cytotoxicity, reduced LDH release and promoted cell viability of HK‐2 cells with LPS‐induced AKI.

KDM2B is also involved in regulating inflammatory responses. KDM2B knockdown resulted in a significant reduction in the expression of several pro‐inflammatory genes, including IL‐6, TNF‐α, and COX‐2. Further experiments demonstrated that the reduction in inflammatory gene expression was due to KDM2B‐mediated epigenetic modifications of the DNA in their promoters, which reduced the accessibility of transcription factors to these regions.^7^ However, in nasal epithelial cells, KDM2B exerts an inhibitory effect on inflammatory response via directly acting on the promoter of inflammatory factor genes IL‐6 and TNF‐α.^21^ Moreover, KDM2B overexpression prevents myocardial injury through reducing the inflammatory response, as KDM2B declines IL‑1β, IL‑6, and TNF‑α in the peripheral blood.^3^ From above, KDM2B has multiple roles in inflammation regulation, it exerts various effects (promote or inhibit) in the production of inflammatory factors in different diseases. In the present study, four typical inflammation molecules (TNF‐α, IL‐1β, IL‐18, and VCAM‐1) were analyzed, which showed all four molecules were increased in LPS‐induced HK‐2 cells, however, their concentration could be declined by KDM2B knockdown, indicating that KDM2B promotes inflammation in sepsis model.

Knockdown of KDM2B resulted in a significant reduction in oxidative stress levels, as evidenced by decreased levels of reactive oxygen species (ROS) and increased quantity of antioxidant enzymes such as catalase and SOD. The reduction in oxidative stress might be associated with KDM2B‐mediated regulation of Nrf2/Keap1 pathway, which plays a critical role in cellular responses against oxidative stress.^22^ In this study, oxidative stress was investigated, and other pathways were also analyzed in HK‐2 cell with KDM2B knockdown, which revealed KDM2B knockdown reduces LPS‐induced oxidative stress in AKI model.

As is reported that knockdown of KDM2B resulted in a significant inhibition of the activation of two major immune signaling pathways, NF‐κB and AP‐1.^3^, ^8^, ^12^ Inhibition of NF‐κB and AP‐1 activation possibly due to KDM2B‐mediated epigenetic modifications of the DNA in the promoter regions of genes involved in these pathways, which reduced the accessibility of transcription factors to these regions.^23^ In addition, to prove that NF‐κB and AP‐1 pathways are necessary for KDM2B in regulating inflammation and oxidative stress, SR11302 or JSH‐23 inhibitor was used to deactivate NF‐κB and AP‐1 pathway, the cell viability, inflammatory and oxidative stress were evaluated, which made a solid conclusion that KDM2B regulated inflammation and oxidative stress through activating NF‐κB and AP‐1 pathways.

This study focused on the phenotypic changes caused by KDM2B overexpression or knockdown, the long‐term effects of KDM2B remains to be elucidated. In addition, KDM2B has been reported to have multiple roles in human diseases, the real efficacy of KDM2B on sepsis needs to be investigated in animal models or in vivo experiments. Thus, more efforts should be made to investigate the effects of KDM2B on sepsis patients, including the long‐term effect, possible side‐effect, and protective rate.

In conclusion, KDM2B knockdown reduced cytotoxicity, inflammation, and oxidative stress in LPS‐induced AKI via inhibiting NF‐κB and AP‐1 pathways. Targeting KDM2B may be a promising strategy for the treatment of sepsis‐associated AKI.

## AUTHOR CONTRIBUTIONS

All authors contributed to the study conception and design. Material preparation and the experiments were performed by Xin Li. Data collection and analysis were performed by Xinyu Tian. The first draft of the manuscript was written by Dongliang Zha ng and all authors commented on previous versions of the manuscript. All authors read and approved the final manuscript.

## CONFLICT OF INTEREST STATEMENT

The authors declare no conflict of interest.

## Supporting information

Supporting Information.

## Data Availability

All data generated or analyzed during this study are included in this published article. The data sets used and/or analyzed during the present study are available from the corresponding author on reasonable request
