# Supplementary figures and images for: KDM2B regulates inflammation and oxidative stress of sepsis via targeting NF‐κB and AP‐1 pathways
Source: Immun Inflamm Dis. 2023 Sep 20;11(9):e985. doi: 10.1002/iid3.985 (PMC10510463; doi:10.1002/iid3.985)

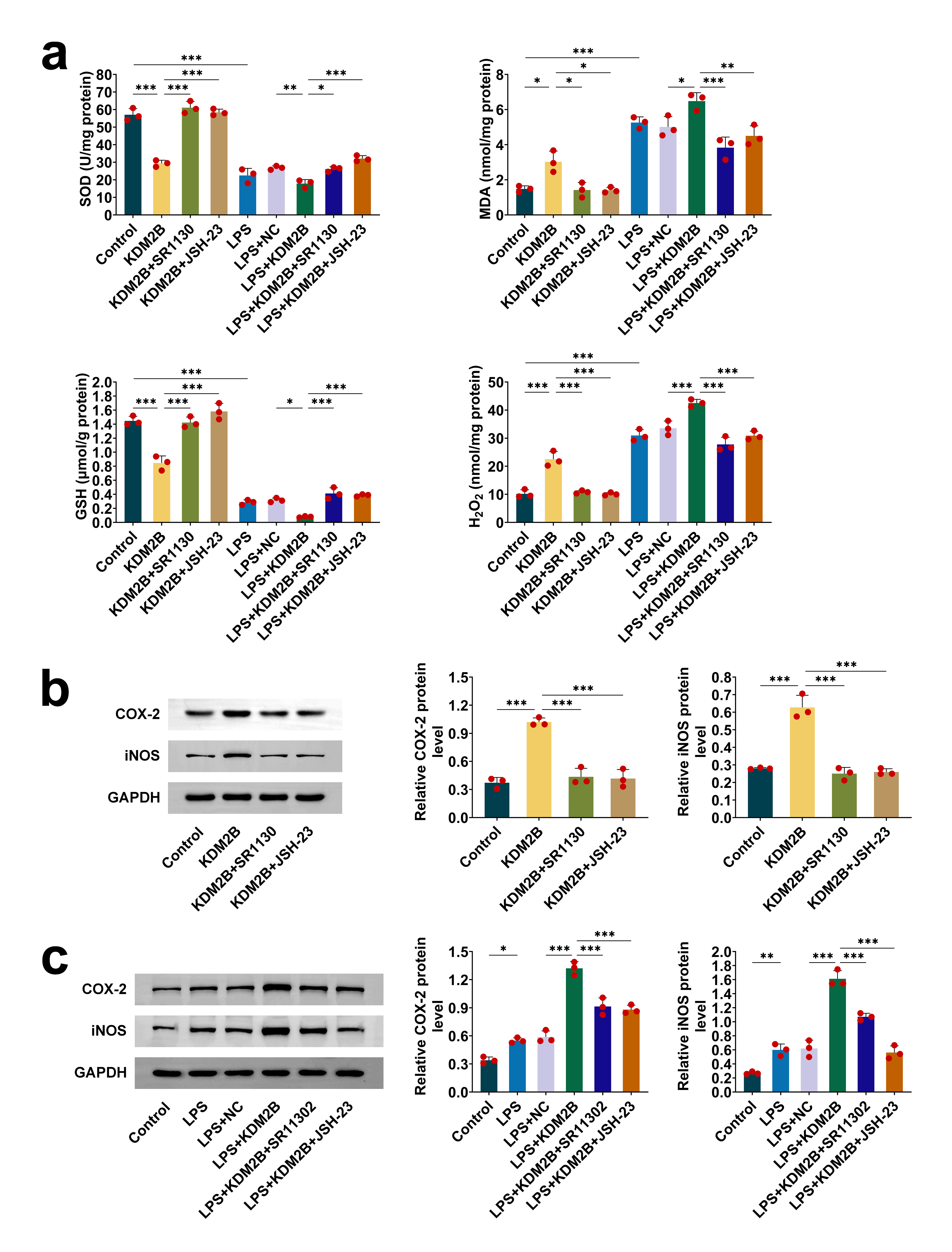

Supplement: Supplementary file 1 — Supporting Information. [file IID3-11-e985-s001.jpg]
